# Machine learning in cardiac stress test interpretation: a systematic review

**DOI:** 10.1093/ehjdh/ztae027

**Published:** 2024-04-17

**Authors:** Dor Hadida Barzilai, Michal Cohen-Shelly, Vera Sorin, Eyal Zimlichman, Eias Massalha, Thomas G Allison, Eyal Klang

**Affiliations:** Sami Sagol AI Hub, ARC, Sheba Medical Center, 31 Emek Ha'ela, Ramat Gan 5262000, Israel; Sami Sagol AI Hub, ARC, Sheba Medical Center, 31 Emek Ha'ela, Ramat Gan 5262000, Israel; Leviev Heart Center, Sheba Medical Center, 31 Emek Ha'ela, Ramat Gan 5262000, Israel; School of Medicine, Tel Aviv University, Tel Aviv, Ramat Aviv 69978, Israel; Department of Diagnostic Radiology, Sheba Medical Center, 31 Emek Ha'ela, Ramat Gan 5262000, Israel; Sami Sagol AI Hub, ARC, Sheba Medical Center, 31 Emek Ha'ela, Ramat Gan 5262000, Israel; School of Medicine, Tel Aviv University, Tel Aviv, Ramat Aviv 69978, Israel; Sheba Medical Center, The Sheba Talpiot Medical Leadership Program, 31 Emek Ha'ela, Ramat Gan 5262000, Israel; Leviev Heart Center, Sheba Medical Center, 31 Emek Ha'ela, Ramat Gan 5262000, Israel; Department of Cardiovascular Medicine, Mayo Clinic, 21 2nd St SW Suite 30, Rochester, MN 55905, USA; Division of Pediatric Cardiology, Department of Pediatric and Adolescent Medicine, Mayo Clinic, 200 1st St SW, Rochester, MN 55905, USA; Sami Sagol AI Hub, ARC, Sheba Medical Center, 31 Emek Ha'ela, Ramat Gan 5262000, Israel; School of Medicine, Tel Aviv University, Tel Aviv, Ramat Aviv 69978, Israel; Department of Diagnostic Radiology, Sheba Medical Center, 31 Emek Ha'ela, Ramat Gan 5262000, Israel; Sheba Medical Center, The Sheba Talpiot Medical Leadership Program, 31 Emek Ha'ela, Ramat Gan 5262000, Israel; Division of Data-Driven and Digital Medicine (D3M), Icahn School of Medicine at Mount Sinai, 1 Gustave L. Levy Place, New York, NY 10029-5674, USA

**Keywords:** Coronary artery disease, Machine learning, Deep learning, Natural language processing, Stress test, Stress electrocardiography, Stress echocardiography, Diagnostic accuracy, Cardiovascular health

## Abstract

Coronary artery disease (CAD) is a leading health challenge worldwide. Exercise stress testing is a foundational non-invasive diagnostic tool. Nonetheless, its variable accuracy prompts the exploration of more reliable methods. Recent advancements in machine learning (ML), including deep learning and natural language processing, have shown potential in refining the interpretation of stress testing data. Adhering to Preferred Reporting Items for Systematic Reviews and Meta-Analyses guidelines, we conducted a systematic review of ML applications in stress electrocardiogram (ECG) and stress echocardiography for CAD prognosis. Medical Literature Analysis and Retrieval System Online, Web of Science, and the Cochrane Library were used as databases. We analysed the ML models, outcomes, and performance metrics. Overall, seven relevant studies were identified. Machine-learning applications in stress ECGs resulted in sensitivity and specificity improvements. Some models achieved rates of above 96% in both metrics and reduced false positives by up to 21%. In stress echocardiography, ML models demonstrated an increase in diagnostic precision. Some models achieved specificity and sensitivity rates of up to 92.7 and 84.4%, respectively. Natural language processing applications enabled the categorization of stress echocardiography reports, with accuracy rates nearing 98%. Limitations include a small, retrospective study pool and the exclusion of nuclear stress testing, due to its well-documented status. This review indicates the potential of artificial intelligence applications in refining CAD stress testing assessment. Further development for real-world use is warranted.

## Graphical Abstract

**Graphical Abstract:**
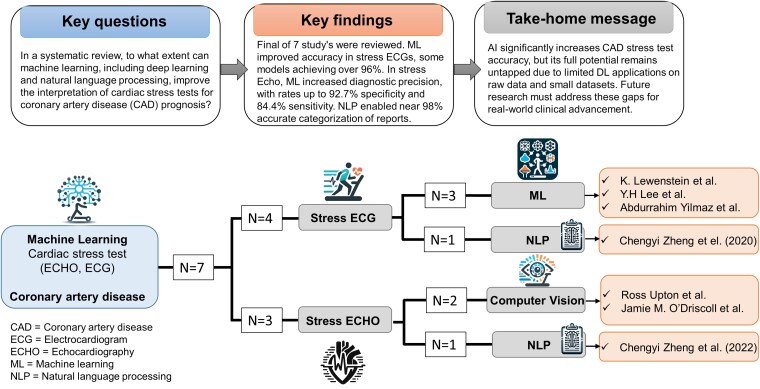

## Introduction

Coronary artery disease (CAD) is a leading global health concern. Exercise stress testing is a cost-effective, non-invasive method to diagnose CAD. It can potentially reduce the need for coronary angiography in negative test cases. However, its diagnostic accuracy can vary based on age, gender, clinical characteristics, CAD prevalence, and the test modality. Identifying which test-negative individuals require urgent coronary angiography remains a question.^1^

Diagnostic accuracy for the detection of CAD can potentially be improved by integrating computer-based image analysis algorithms.^2^ With recent advancements in computational power and vast data sets, machine learning (ML) has become a pivotal diagnostic tool in healthcare. Deep learning (DL) algorithms, an ML subset, can interpret patterns in biomedical data.^3^ Another ML technology suitable for this purpose is natural language processing (NLP), which is a computational technique for analysing free text. Its growing use in healthcare is transforming text extraction and processing tasks.^4^

Deep learning has proven valuable in analysing resting electrocardiograms (ECGs) and detecting arrhythmias such as atrial fibrillation^5^ and ventricular tachycardia.^6^ Moreover, these algorithms have pinpointed patterns indicating cardiac conditions such as valvular diseases, cardiac amyloidosis, and hypertrophic cardiomyopathy.^7–10^ Deep learning can also be used for the analysis of stress ECG and stress echocardiography.

Our study aims to review the literature on ML applications in stress echocardiography and stress ECG.

## Methods

### Search strategy

We conducted a systematic review following the Preferred Reporting Items for Systematic Reviews and Meta-Analyses (PRISMA) statement for critical appraisal and data extraction.^11^ We searched for peer-reviewed original articles that evaluated the performance of ML models in predicting CAD from cardiac stress testing (stress ECG and stress echocardiography).

The search was performed using Medical Literature Analysis and Retrieval System Online, Web of Science, and the Cochrane Library from inception to 19 May 2023, using the following keywords: ‘artificial intelligence’, ‘deep learning’, ‘machine learning’, ‘convolutional neural networks’, ‘stress test’, ‘stress ECG’, ‘exercise test’, ‘stress echocardiography’, ‘Pharmacologic Stress Test’, ‘chemical Stress Test’, ‘Adenosine Stress Test’, and ‘Dobutamine Stress Test’. We excluded non-English articles and conference abstracts and reviewed the references of the included papers. Supplementary material online, *S1* and *S2* represent the PRISMA checklist and the complete search strategy, respectively. Our study was registered with PROSPERO (ID: CRD42023426438).

### Study selection

**Figure 1:**
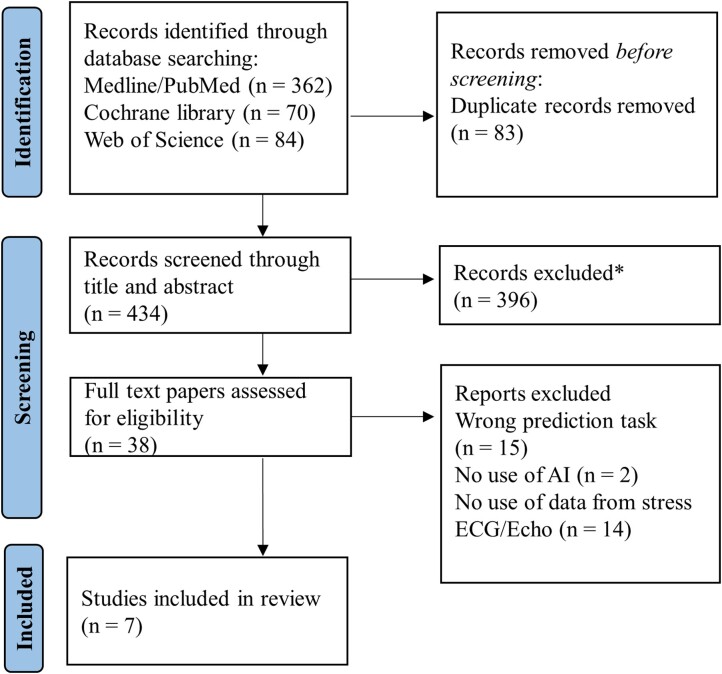
A flow chart of the sequential flow of the search and inclusion process followed in the study. The study adhered to the guidelines outlined in the Preferred Reporting Items for Systematic Reviews and Meta-Analyses for its reporting. *Studies were excluded based on title and abstract screening, focusing on the use of machine learning in cardiac stress electrocardiogram and echocardiogram interpretation.

Two authors (D.H.B. and M.C.-S.) independently screened titles and abstracts according to the eligibility criteria, which were predetermined and documented in our study protocol registered with PROSPERO (ID: CRD42023426438). Full-text articles were evaluated when inclusion or exclusion criteria were inconclusive. Any disagreements between the two reviewers were resolved by a third reviewer (E.K.). Subsequently, the two authors independently assessed the full-text studies. *Figure 1* provides a flow chart detailing the screening and inclusion procedure.

### Data extraction

We used a standardized data extraction sheet to collect relevant data from the reviewed, including year of publication, journal name, and country. We also summarized the input type, prediction task, application, number of participants, and ML architecture information.

### Outcomes and evaluation metrics

We identified primary outcomes that centred on measuring the performance of ML models in the detection of CAD, including sensitivity, specificity, positive predictive value, negative predictive value, and area under the curve.

Additionally, we explored secondary outcomes, encompassing various aspects such as the ML architecture used, clinical relevance, reduction in false-positive rates, and the nuanced understanding of cardiac function.

### Risk of bias

In evaluating the quality and diagnostic accuracy of the studies under review, the Quality Assessment of Diagnostic Accuracy Studies-2 (QUADAS-2) tool was used. This comprehensive tool systematically assesses the risk of bias and applicability across four critical domains: patient selection, index test, reference standard, and flow and timing. The methodology of each study categorized the potential for bias and applicability concerns as either ‘high’, ‘low’, or ‘some concerns’.

## Results

### Study selection and characteristics

Overall, seven studies were included in this review, all published between 2001 and 2023. Four studies evaluated artificial intelligence (AI) applications for stress ECG and three for stress echocardiography.

*Table 1* provides a detailed overview of the primary characteristics of the articles included in the review. In *Table 2*, algorithm types and performance are detailed.

**Table 1:** Basic characteristics of publications reporting on deep learning used in cardiac stress testing

Title	Authors	Publication date	Country	Journal
Machine learning of treadmill exercise test to improve selection for testing for coronary artery disease	Lee *et al*.^12^	2022	Taiwan	*Atherosclerosis*
Automated echocardiographic detection of severe coronary artery disease using artificial intelligence	Upton *et al*.^13^	2022	UK	*JACC*: *Cardiovascular Imaging*
Automated interpretation of stress echocardiography reports using natural language processing	Zheng *et al*.^14^	2022	USA	*European Heart Journal – Digital Health*
Automated identification and extraction of exercise treadmill test results	Zheng *et al*.^15^	2020	USA	*Journal of the American Heart Association*
Radial basis function neural network approach for the diagnosis of coronary artery disease based on the standard electrocardiogram exercise test	Lewenstein^16^	2001	Poland	*Medical & Biological Engineering & Computing*
Left ventricular assessment with artificial intelligence increases the diagnostic accuracy of stress echocardiography	O’Driscoll *et al*.^17^	2022	UK, US	*European Heart Journal Open*
Machine learning approach on high-risk treadmill exercise test to predict obstructive coronary artery disease by using P, QRS, and T wave’ features	Yilmaz *et al*.^18^	2023	Turkey	*Current Problems in Cardiology*

**Table 2:** Publications reporting on machine learning used in cardiac stress testing

Citation	Input	Prediction task	Application	Number of participants	Architecture	Sensitivity	Specificity	PPV	NPV	AUC	Percentage of positive tests	Model assessment	Gold standard	Non-AI standard of interpretation	Feature importance
K. Lewenstein	Stress ECG	CAD, specific vessel	Radial basis function neural network used for CAD diagnosis based on the results of the traditional ECG stress test	776	RBF	97.3	97.8	—	—	—	61.7%	Leave-one-out cross-validation, generalized cross-validation	Invasive coronary angiography; >50% stenosis in any vessel	EHR	Not performed
C. Zheng *et al*. (2020)	Stress ECG reports	CAD	NLP algorithm designed to categorize stress ECG reports into normal, ischaemic, non-diagnostic, and equivocal categories	5214	NLP (rule-based)	96.4	94.8	87.1	98.6	—	5.9%	Separate internal validation data set (*n* = 105)	Stress ECG reports	Cardiologists and emergency medicine physicians conducted *blind* evaluations of randomly selected reports	Not performed
Y.-H. Lee	Stress ECG	CAD	Prediction of CAD by ML models trained with stress ECG and clinical features	2325	Five ML models: SVM, KNN, LR, RF, and XGboost	0.85 ± 0.06	0.45 ± 0.05	0.45 ± 0.02	0.85 ± 0.05	0.74 ± 0.04	34.9%	30% of holdout test set (*n* = 698). Results were compared with the conventional exercise ECG test report	Invasive coronary angiography; stenosis >50% in LM artery or >70% in other arteries	Interventional cardiologists	Feature selection involved recursive elimination with cross-validation, selecting 30 out of the 93 features such as: exercise performance, haemodynamics, and ST-segment changes; The study did not report their relative importance
A. Yilmaz	Stress ECG	Obstructive CAD	Prediction of CAD using time and amplitude features of P, QRS, and T waves of V5	294	5 ML models: GP classifier, SVM, kNN, MLP, and XGboost	67.24 ± 12.61%	84.62 ± 7.30%	74.32 ± 9.39%	87.79 ± 7.94%	0.78 ± 0.06	32%	5-fold cross-validation on the entire cohort. Results also compared with the performance of cardiologists on V5 signal	Invasive coronary angiography; >70% in any coronary artery	Experts cardiologists	Recursive feature elimination, feature importance, and t-SNE were used to select final 23 signal features manually, including information on P, QRS, and T waves. The study did not report their relative importance
R. Upton *et al*.	Stress ECHO Dobutamine (87.2%)	Severe CAD	An automated image processing pipeline to extract novel geometric and kinematic features from stress echo. An ensemble ML classifier was trained, using the extracted features, to identify patients with severe CAD	732	CNN (EchoGo Core 1.0)	84.4 (73.9–95.0%)	92.7 (87.8–97.6%)	—	—	0.93	27%	Cross-validation on the entire cohort and external validation on a separate retrospective data set	Invasive coronary angiography; stenosis >50% in LM artery or >70% in other arteries	*Blinded a*djudication committee, comprising at least one cardiologist	Feature analysis revealed equal contribution across AUROCs with 20 features from the A4C view, 2 from the A2C, and 9 from the SAX, focusing on apical lateral and mid anterolateral sections
C. Zheng *et al*. (2022)	Stress ECHO reports Dobutamine (40.2%)	CAD	An automated NLP algorithm to abstract stress echo reports and classify the overall results into normal, non-diagnostic, infarction, and ischaemia categories according to the various stress echo parameters	6346	NLP (rule-based)	95.7 (90.9–98.4)	98.6 (96.9–99.5)	95.7 (91.0–98.0)	98.6 (96.9–99.3)	—	12.8%	Separate internal validation data set (*n* = 150)	Stress echo reports	Cardiologists conducted *blind* evaluations of randomly selected reports	Not performed
J.M. O’Driscoll *et al*.	Stress ECHO Dobutamine (72%)	CAD	AI automated contouring of the endocardial borders, automated identification of the end diastolic and systolic frames for the calculation of LVEF and GLS during stress echo for CAD prediction	500	CNN (EchoGo Core 1.0) and multivariable risk models	76	94	72	95	AI-LVEF = 0.86; AI-GLS = 0.82	14.8%	External validation was conducted with a cohort of 517 participants across seven distinct medical centres	Invasive coronary angiography; stenosis >50% in LM artery or >70% in other arteries	*Blinded* adjudication committee, comprising at least one cardiologist	Not performed

AI, artificial intelligence; AUC, area under the curve; CAD, coronary artery disease; CNN, convolutional neural networks; GLS, global longitudinal strain; KNN, *K*-nearest neighbours algorithm; LR, logistic regression; LVEF, left ventricle ejection fraction; ML, machine learning; NLP, natural language process; NPV, negative predictive value; PPV, positive predictive value; RBF, radial basis function; RF, random forest; stress ECG, stress electrocardiography; stress ECHO, stress echocardiography; SVM, support vector machine; XGboost, extreme gradient boosting.

### Risk of bias

**Figure 2:**
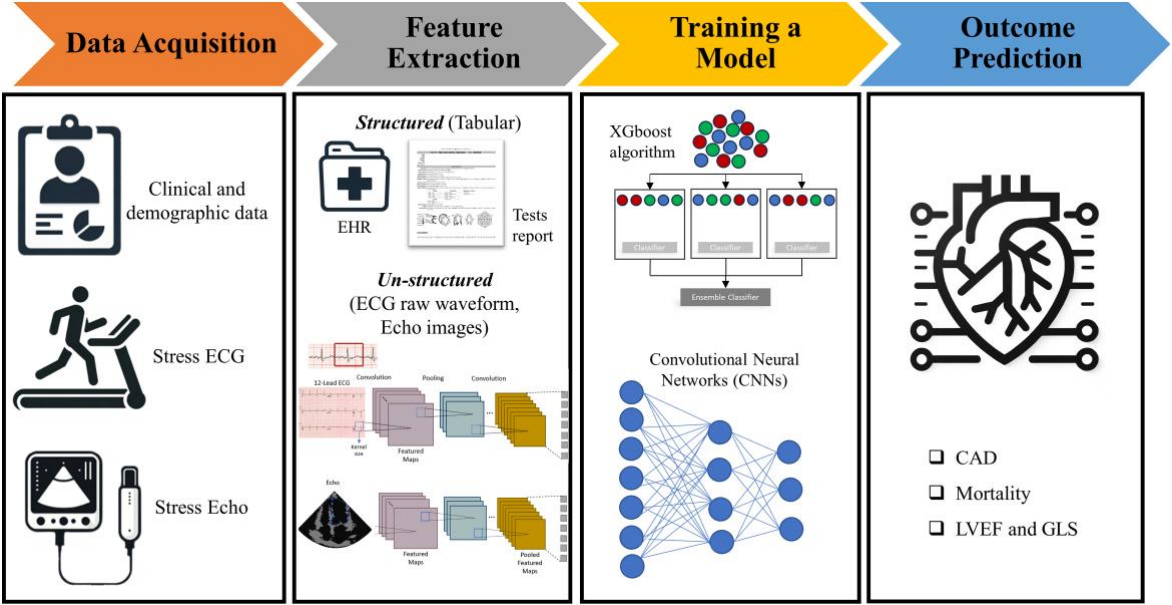
A workflow diagram illustrating the process of utilizing machine learning for the interpretation of stress echocardiography and stress electrocardiogram. The initial step involves the acquisition of comprehensive data sources, including clinical information, stress electrocardiogram, and echocardiography. Once gathered, these data sets undergo a crucial feature extraction process to distil essential indicators and patterns. Following this, a machine-learning model is trained on the extracted features, enhancing its understanding and capability. The culmination of this process is the model’s ability to predict cardiac outcomes with increased accuracy and insight. CAD, coronary artery disease; CNN, convolutional neural networks; EHR, electronic health record; GLS, global longitudinal strain; Stress ECG, stress electrocardiography; stress ECHO, stress echocardiography; LVEF, left ventricle ejection fraction; XGboost, Extreme Gradient Boosting.

**Figure 3:**
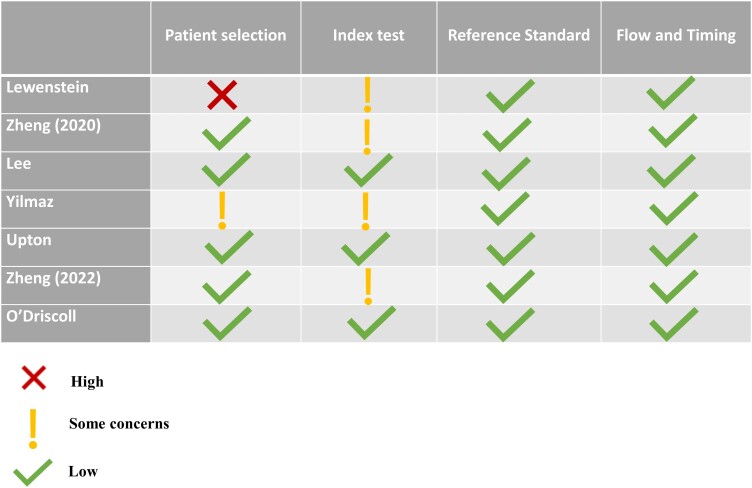
Results of the quality assessment for diagnostic accuracy studies 2.

Using the QUADAS-2 tool, we evaluated the risk of bias in multiple domains: patient selection, the index test, the reference standard, and flow and timing as depicted in *Figure 2*. With regard to patient selection, most studies conducted across multiple centres adhered to rigorously defined inclusion and exclusion criteria. However, uncertainties arose in two instances: Lewenstein’s^16^ exclusion of female participants and Yilmaz *et al*.’s^18^ focus on high-risk patients (Duke treadmill score <−10) from a single cardiology clinic. No studies employed a case–control design. In the evaluation of the index test and reference standard, the majority of the included studies demonstrated a range of low-to-moderate concerns regarding the risk of bias. This observation is attributed to well-defined methodologies alongside a consistent application of index and reference standards—namely, standardized protocols for stress echocardiography and ECG interpreted by AI, with invasive coronary angiography serving as the gold standard for CAD diagnosis. Notably, two studies designated the stress ECG/echocardiography report as the ground truth, leveraging NLP for report classification, aligned with their primary objectives. With regard to the validation of the ML models, Lewenstein^16^ and Yilmaz *et al*.^18^ opted for internal cross-validation, and Zheng *et al*.^14,15^ employed a relatively small, separate internal validation data set, methods considered appropriate for small cohort validation yet bearing potential bias risks. Conversely, Upton *et al*.^13^ and O’Driscoll *et al*.^17^ adopted a more comprehensive approach by integrating both internal cross-validation and external validation on distinct data sets. In terms of flow and timing, given the AI-centric approach, traditional patient flow concerns were less applicable, generally indicating low bias risk with consistent testing processes and timing. Overall, while many studies exhibited a minimal risk of bias and concerns regarding applicability, specific instances underscore the importance of refining patient selection strategies in future investigations (*Figure 3*).

### Stress electrocardiogram

The integration of ML models in stress ECG has shown a promising success rate, drawing from diverse study populations in the USA, Poland, Taiwan, and Turkey. Among these studies, two out of four utilized traditional ML algorithms, and one used a radial neural network for CAD diagnosis, considering coronary angiography as the gold standard for confirmation. Studies by Zheng *et al*.^15^ and Lewenstein^16^ demonstrated sensitivity levels exceeding 96% and specificity surpassing 94% for detecting CAD, surpassing human-based interpretation rates of 68 and 77%, respectively.^19^

Lee *et al*.^12^ used various ML algorithms and reduced the false-positive rate of stress ECG examinations from 76.3 to 55% compared with conventional testing methods. Notably, their random forest model performed better on men (AUC of 0.72) than on women (AUC of 0.68) ECG examinations.

Yilmaz *et al*.^18^ developed five ML models that use time and amplitude features of the P, QRS, and T waves in V5. All five models outperformed cardiologists’ estimates in identifying obstructive CAD.

Zheng *et al*.^15^ developed an accurate strategy to facilitate large-scale outcome studies using an NLP algorithm. The model not only accurately identified reports with abnormal stress test results but also validated a strong association with 30-day acute myocardial infarction or mortality.

### Stress echo

In the domain of stress echocardiography interpretation with ML, several studies have shown high metrics (*Tables 1* and *2*).

Upton *et al*.^13^ developed an automated pipeline using Convolutional Neural Networks (CNNs) to extract features from stress echocardiography examinations. These features were used to train an ensemble ML classifier to identify patients with severe CAD. Their ML model achieved a specificity of 92.7% and a sensitivity of 84.4% during cross-validation. Incorporating AI classifications into clinical practice improved inter-reader agreement, confidence, and sensitivity, enhancing CAD detection by 10%. The model’s high accuracy was validated in a separate study by O’Driscoll *et al*.^17^ with an AUC of 0.93. The authors explored AI’s role in calculating left ventricular ejection fraction and global longitudinal strain, emphasizing the potential of AI in refining stress echo interpretation.

Zheng *et al*.^14^ employed NLP to extract and categorize stress echocardiography reports automatically. Their NLP algorithm demonstrated 98.6% specificity, 95.7% sensitivity, and an *F*-score of 0.957 in identifying ischaemia.

## Discussion

A persistent clinical challenge remains the identification of individuals necessitating urgent coronary angiography, particularly among those with negative stress test results.^1^ Our systematic review explores utilizing ML techniques, including DL and NLP, for stress echocardiography and ECG. While DL algorithms excel in analysing raw stress test data, NLP offers another advantage in processing and interpreting the textual data generated by human experts. This distinction highlights their complementary roles in AI-driven cardiac diagnostics.

### Deep learning in raw data analysis

Deep learning algorithms’ ability to uncover complex patterns in stress test data represents a significant improvement in CAD detection, offering precision beyond conventional interpretation. However, the stress ECG studies reviewed used traditional ML algorithms on structured data such as measurements and demographics and not DL on raw signals. This gap, coupled with the challenge of relatively small data sets, underscores an untapped area for future research. Notably, the lack of digitally available retrospective data serves as a bottleneck, delaying the development of DL studies on stress ECG raw data. Although these algorithms improved the identification of true negatives and addressed concerns related to false positives, issues such as gender disparities in algorithm performance, as observed by Lee *et al*.,^12^ need addressing. Conversely, for stress echocardiography, DL algorithms were applied to raw data, automating interpretations. This review emphasizes AI’s potential in enhancing clinical decision-making, boosting physician confidence in examination interpretation, and streamlining patient management. Artificial intelligence–based test interpretations are conducted simultaneously and follow consistent principles, minimizing external biases inherent in conventional human-based interpretation.

### Natural language processing in interpreting textual data

In contrast to DL’s focus on raw signals, NLP technologies excel at extracting and interpreting valuable insights from the textual reports generated by clinicians. This application of AI addresses a need for accurate categorization and analysis of stress test reports, as shown in Zheng *et al*.,^14,15^ streamlining diagnostic processes and supporting clinical decision-making. By automating the interpretation of textual data, NLP facilitates a more efficient review of patient reports, identifying reporting errors and quality gaps that could impact patient care. Moreover, integrating NLP into electronic health record systems promises to improve the accessibility and quality of medical information, thereby enhancing patient outcomes and healthcare equity.

Despite the promising applications of AI in stress testing, considering the practical challenges of implementing these technologies in real-world clinical settings is crucial. Challenges, such as ensuring data quality and availability, achieving model interpretability, integrating AI tools into existing healthcare systems, navigating regulatory landscapes, and addressing potential biases to ensure generalizability (including continuous monitoring of data and performance drift to provide ‘responsible AI’), are pivotal. Another challenge is the resistance from clinical staff to these tools, which must be seamlessly integrated into routine workflows and include an explainability component to create transparency in the machine’s decision-making process; they cannot remain a ‘black box’. As demonstrated by Lee *et al*.^12^ and Upton *et al*.,^13^ the algorithms can replicate disparities in healthcare, as they did not account for gender, race, and ethnicity during the development of the model.^12,13^ The algorithms’ accuracies are variable, and they still require supervision and validation. Moreover, integrating these algorithms into workflows introduces cybersecurity risks.^20^ Addressing these challenges requires a concerted effort from researchers, clinicians, legal teams, and technology developers to substitute an environment where AI can be safely and effectively integrated into cardiac diagnostics. Future research should focus on developing interpretable and bias-free models, standardized data collection protocols, and ethical guidelines for AI use, ensuring these innovative tools can truly improve patient care in diverse clinical settings.

This study has several limitations. First, the number of studies included in this review is relatively small, and all the studies are of a retrospective nature. Nuclear stress testing was excluded from the scope of our review due to its well-established and extensively researched nature, characterized by numerous publications and reviews.^21–23^ Among the seven studies, four were conducted in multicentre settings, and three explicitly emphasized the need for external validation of their models. Secondly, the heterogeneity observed in patient populations, data sources (including raw data from stress ECG, stress echocardiography, and text analysis), ML architectures, and even the variability in the objectives of ML applications (ranging from the classification of test reports and automation of diagnosis to ventricular function assessment and prognosis prediction) among the reviewed studies, barred us from conducting a meta-analysis. Future research must adopt standardized methodologies to better support meta-analysis in this rapidly evolving field. Finally, as AI in cardiology is rapidly advancing, there exists the possibility of manuscripts and applications being published after this review. There is a need for larger, more diverse, and prospective studies to validate these AI applications and ensure their applicability and generalizability in clinical practice. Future trials like PROTEUeS (ClinicalTrials.gov Identifier: NCT05028179) exemplify this endeavour.

## Conclusions

This review indicates AI application potential in refining CAD stress testing assessment. Further development for real-world use is warranted.

## Supplementary Material

ztae027_Supplementary_Data

## Data Availability

The data underlying this systematic review are derived from publicly available sources. All articles and data sets analysed are available in PubMed, Web of Science, and the Cochrane library and can be accessed through the references listed in the manuscript.
